# Arginase and Arginine Dysregulation in Asthma

**DOI:** 10.1155/2011/736319

**Published:** 2011-04-26

**Authors:** Renée C. Benson, Karen A. Hardy, Claudia R. Morris

**Affiliations:** ^1^Bay Area Pediatric Pulmonary Medical Corporation, Children's Hospital & Research Center Oakland, Oakland, CA 94609, USA; ^2^Department of Emergency Medicine, Children's Hospital & Research Center Oakland, Oakland, CA 94609, USA

## Abstract

In recent years, evidence has accumulated indicating that the enzyme arginase, which converts L-arginine into L-ornithine and urea, plays a key role in the pathogenesis of pulmonary disorders such as asthma through dysregulation of L-arginine metabolism and modulation of nitric oxide (NO) homeostasis. Allergic asthma is characterized by airway hyperresponsiveness, inflammation, and remodeling. Through substrate competition, arginase decreases bioavailability of L-arginine for nitric oxide synthase (NOS), thereby limiting NO production with subsequent effects on airway tone and inflammation. By decreasing L-arginine bioavailability, arginase may also contribute to the uncoupling of NOS and the formation of the proinflammatory oxidant peroxynitrite in the airways. Finally, arginase may play a role in the development of chronic airway remodeling through formation of L-ornithine with downstream production of polyamines and L-proline, which are involved in processes of cellular proliferation and collagen deposition. Further research on modulation of arginase activity and L-arginine bioavailability may reveal promising novel therapeutic strategies for asthma.

## 1. Introduction

Since the identification of nitric oxide as a bioactive molecule involved in the pathogenesis of pulmonary disorders, much research has focused on the importance of the nitric oxide synthase pathway involving conversion of L-arginine to NO and L-citrulline. More recently, the arginase pathway involving catabolism of L-arginine to L-ornithine and urea has garnered attention for its potential role in arginine dysregulation and alteration of nitric oxide metabolism, with implications for the pathogenesis of airway diseases such as asthma.

## 2. Asthma and Arginine Dysregulation

Allergic asthma is a chronic obstructive disease of the airways characterized by airway hyperresponsiveness, inflammation, and remodeling. Inhalation of allergen immediately induces the early asthmatic reaction (EAR) which involves cross-linking of IgE by allergen, followed by activation of cells bearing IgE receptor (predominantly mast cells and basophils) with subsequent release of cytokines, proteases, and proinflammatory mediators such as histamine [1, 2]. This rapid inflammatory cascade leads to vasodilation and mucosal edema, mucus secretion, and contraction of airway smooth muscle. The late asthmatic reaction (LAR) is an intense IgE-mediated inflammatory response dominated by infiltration of eosinophils and mononuclear cells that begins three to nine hours after allergen challenge and is correlated with intensity of associated bronchial hyperresponsiveness [3, 4]. By 24–48 hours, T_H_2 cells elaborating cytokines IL-4, IL-5, GM-CSF, and IL-13 can be found infiltrating the inflamed airway, leading to further IgE production, induction of vascular adhesion molecules, promotion of chemotaxis, and eosinophil and macrophage activation [2]. Ongoing exposure to environmental allergens contributes to chronic inflammation and may result in airway remodeling characterized by hypertrophy of submucosal gland mass, smooth muscle cell hyperplasia, and basement membrane thickening due to subepithelial deposition of collagen [1, 5]. Such remodeling may lead to progressive loss of lung function over time [1, 6, 7]. Recent evidence demonstrates that arginase may play a role in arginine dysregulation which contributes to the pathogenesis of asthma through effects on altered NO metabolism.

## 3. Nitric Oxide Metabolism and Airway Function

Nitric oxide (NO) has been well described in the literature as an important signaling molecule involved in regulation of many mammalian physiologic and pathophysiologic processes, particularly in the lung [8, 9]. NO plays a role in regulation of both pulmonary vascular tone as well as airway bronchomotor tone through effects on relaxation of smooth muscle. In addition, NO participates in inflammation and host defense against infection via alterations in vascular permeability, changes in epithelial barrier function and repair, cytotoxicity, upregulation of ciliary motility, altered mucus secretion, and inflammatory cell infiltration [10, 11]. These multiple functions of NO have been implicated in the pathogenesis of chronic inflammatory airway diseases such as asthma. 

NO is produced by a family of nitric oxide synthases (NOSs) that metabolize L-arginine through the intermediate N-hydroxy-L-arginine (NOHA) to form NO and L-citrulline using oxygen and NADPH as cosubstrates. Three NOS mammalian isoenzymes have been identified with varying distributions and production of NO. Neuronal NOS (nNOS or NOS I) and endothelial (eNOS or NOS III) are constitutively expressed (cNOS) in airway epithelium, inhibitory nonadrenergic noncholinergic (iNANC) neurons, and airway vasculature endothelial cells. Their activity is regulated by intracellular calcium, with rapid onset of activity and production of small amounts of NO on the order of picomolar concentrations. Inducible NOS (iNOS or NOS II) is transcriptionally regulated by proinflammatory stimuli, with the ability to produce large amounts (nanomolar concentrations) of NO over hours [11, 12]. 

iNOS is known to be upregulated in asthmatic lungs, and increased levels of exhaled NO are well described in asthma patients [13, 14]. Supplemental oral or inhaled L-arginine increases exhaled NO in both normal and asthmatic subjects, indicating that the bioavailability of L-arginine for NOS determines NO production within the airways [15–17]. In guinea pig tracheal preparations, L-arginine has been shown to inhibit airway hyperresponsiveness to methacholine and to increase iNANC nerve-mediated airway smooth muscle relaxation via increased production of NOS-derived NO [18, 19]. Conversely, inhibition of NOS-derived NO by N (G-) nitro-L-arginine methyl ester (L-NAME) amplifies bronchoconstriction in guinea pigs [20].

## 4. L-Arginine Metabolism Determines NO Production

As the only substrate for NOS, L-arginine bioavailability plays a key role in determining NO production and is dependent on pathways of biosynthesis, cellular uptake, and catabolism by NOS and arginase. Biosynthesis of the semiessential amino acid occurs in a stepwise fashion. L-glutamine and L-proline are absorbed from the small intestine and converted to L-ornithine. L-citrulline is then synthesized from L-ornithine by ornithine carbamoyltransferase (OTC) and carbamoylphosphate synthetase 1 (CPS1) in hepatocytes as part of the urea cycle, as well as in the intestine. L-arginine is produced from L-citrulline by cytosolic enzymes argininosuccinate synthetase 1 (ASS1) and argininosuccinate lyase (ASL). When L-arginine is subsequently metabolized to NO via NOS, L-citrulline is again produced and can be used for recycling back to L-arginine, which may be an important source of L-arginine during prolonged NO synthesis by iNOS [12]. 

The primary source of L-arginine for most cells is cellular uptake via the Na-independent cationic amino acid transporter (CAT) proteins of the y^+^-system. In particular, upregulation of CAT-2B has been associated with increased L-arginine uptake under conditions of iNOS induction stimulated by proinflammatory mediators lipopolysaccharide (LPS) and interferon-*γ* (IFN-*γ*) [21–26]. Ablation of the CAT-2 gene is associated with impaired iNOS-mediated NO synthesis in macrophages and astrocytes, which implies an important role of CAT-2 in uptake of L-arginine substrate for iNOS [27, 28]. 

L-arginine uptake via the y^+^-system can be inhibited by other amino acids such as L-ornithine and L-lysine, as well as by polycations such as eosinophil-derived major basic protein (MBP) and poly-L-arginine [21, 29, 30]. MBP inhibition of L-arginine uptake was associated with decreased NO synthesis in rat alveolar macrophages and tracheal epithelial cells, most likely related to reduced L-arginine availability [24]. In addition, airway hyperresponsiveness to methacholine has been shown to increase in rats and guinea pigs after treatment with poly-L-arginine, related to attenuation of epithelial NO production. Treatment with combined poly-L-arginine and the antagonist polyanion heparin restored L-arginine uptake and NO production and reversed airway hyperresponsiveness [31–33].

## 5. Arginase and Catabolism of L-Arginine

More recently, focus has turned to the pathway of L-arginine catabolism by arginase as important in regulating endogenous NO production, with implications for airway function in lung diseases such as asthma. Arginase is a urea cycle enzyme that catalyzes the hydrolysis of L-arginine to urea and L-ornithine. Both Arginase I and II isoforms are constitutively expressed in the airways; Arginase I is additionally located in the cytosol of hepatic cells, while arginase II is mitochondrial and extrahepatic [12, 34]. While the affinity (Km) of L-arginine for arginase is in the low micromolar range compared to the low millimolar range for NOS, substrate competition does occur between arginase and NOS because the Vmax of arginase is 1000-fold higher [35–37]. By competing for a common substrate, arginase reduces the bioavailability of L-arginine for NOS, therefore limiting NO production. Specific arginase inhibitor N-hydroxy-nor-L-arginine (nor-NOHA) has been shown to attenuate methacholine-induced constriction of guinea pig trachea and to increase iNANC-mediated relaxation of tracheal smooth muscle preparations, which is consistent with increased NO production through NOS under conditions of arginase inhibition [19, 38] (see Table 1 for list of pharmacologic enzyme inhibitors). NOS can also inhibit arginase activity through NOHA, the intermediate in NO synthesis [39]. Arginase product L-ornithine may also play a role in regulating availability of L-arginine to NOS through competitive inhibition of arginase [37, 40, 41] as well as inhibition of L-arginine uptake by CATs of the y^+^-system [21, 29, 30]. L-ornithine also serves as a substrate for ornithine decarboxylase (ODC), which synthesizes polyamines involved in promotion of cell growth and repair, and for ornithine aminotransferase (OAT), leading to formation of L-proline which is required for collagen synthesis [12].

## 6. T_H_1/T_H_2 Regulation of Arginase and iNOS in Asthma

Allergic asthma is clearly a complicated inflammatory disease involving many different stages, cell types, cytokines, and mediators which remain incompletely understood. Depending on the stage, different inflammatory mediators are released from the various cell types, with implications for NOS/arginase activity and airway function. Mast cells and basophils are involved in the EAR and secrete histamine and TNF-*α*. Eosinophils play an important role in the LAR and contribute to ongoing inflammation through secretion of cytotoxic major basic protein and eosinophilic cationic protein. T cells are also key players in the pathophysiology of asthma, with the T_H_1/T_H_2 balance well described as being weighted towards the T_H_2 pathway. T_H_1 cells (which secrete IL-2, IFN-*γ*, and TNF-*β*) are involved in macrophage activation and cell-mediated immunity. T_H_2 cells (which secrete IL-4, IL-5, IL-10, and IL-13) are involved in mast cell and eosinophil activation, and humoral immunity [2, 51]. 

The balance of iNOS versus arginase activity and level of NO production in the airway may be related to the balance of T_H_1/T_H_2 cytokines during the inflammatory cascade. Experimental models of asthma have demonstrated that iNOS is induced by proinflammatory T_H_1 cytokines released from mast cells (such as TNF-*α*, IFN-*γ*, and IL-1) after allergen challenge and during the LAR [9, 14, 52, 53]. Arginase activity is induced (and iNOS suppressed) by T_H_2 cytokines IL-4 and IL-10 in murine macrophages, although IL-4 does not induce arginase in human macrophages unless combined with agents that increase cAMP [54–56]. Arginase activity increases following challenge with allergens such as ovalbumin, *Dermatophagoides farinae*, and *Aspergillus fumigatus* in guinea pig tracheal preparations, and in mouse and rat models of allergic asthma [44, 57–61]. Gene expression studies have also shown induction of arginase I more than arginase II gene expression in mouse models of allergen-challenged lungs and T_H_2 cytokine-mediated lung inflammation [59, 62–65].

## 7. Altered NO Metabolism and Airway Hyperresponsiveness

In asthmatic patients as well as experimental models of asthma, increased NO production occurs in the airways related to upregulation of iNOS by proinflammatory cytokines after allergen challenge and during the LAR [9, 14, 52, 53]. This upregulation of iNOS in airway epithelial cells and inflammatory cells is associated with airway eosinophilia, airway hyperresponsiveness (AHR), and increased NO in exhaled air [9, 16, 66–70]. Anti-inflammatory treatment with corticosteroids reduces all of these markers [71, 72]. The increased production of NO may actually be a protective mechanism to maintain airway tone in the setting of inflammatory changes. Studies of iNOS knockout mice have demonstrated increased airway inflammation and AHR compared to wild-type mice [46], whereas mice that overexpress iNOS demonstrate increased exhaled NO and decreased AHR without airway inflammation [73]. 

Airway inflammation in asthma may not be the result of increased NO production itself, but rather due to the formation of the proinflammatory oxidant peroxynitrite from reaction of NO with superoxide anions in the airway. Peroxynitrite activates eosinophils, increases MUC5AC expression, increases microvascular permeability, induces airway epithelial damage, and augments airway smooth muscle contraction [74–77]. Airway epithelial cells and inflammatory cells from bronchial biopsies of asthmatics as well as allergen-challenged guinea pigs demonstrate increased nitrotyrosine immunostaining (a marker for peroxynitrite nitration of protein tyrosine), which is also correlated with increased exhaled NO, iNOS expression, AHR, and eosinophilic inflammation [75, 78, 79]. The AHR observed after allergen challenge and the LAR may be the result of increased peroxynitrite formation [74, 80].

## 8. Arginine Dysregulation Contributes to Airway Hyperresponsiveness

In contrast to the increased NO production seen during the LAR, the increased AHR seen after the EAR may paradoxically involve NO deficiency within the airways related to reduced bioavailability of L-arginine to both cNOS and iNOS. 

In guinea pig models of acute allergic asthma, exhaled NO drops during the allergen-induced EAR, and iNOS is not detected until the LAR, indicating that decreased cNOS production of NO may contribute to subsequent AHR [67, 81–83]. Table 2 describes the relative activity of NOS and arginase with respect to the various stages of allergic asthma. In patients with severe asthma and evidence of AHR treated with corticosteroids, inhaled NOS inhibitor N^G^-monomethyl-L-arginine (L-NMMA) failed to potentiate bradykinin-mediated bronchoconstriction. The authors concluded that this effect reflected corticosteroid downregulation of iNOS, with reduced cNOS-derived NO leading to failure of bronchoprotection and increased AHR [48]. 

Reduced L-arginine bioavailability to cNOS may be responsible for the NO deficiency seen after the allergen-induced EAR, as animal studies supplementing L-arginine have shown decreased airway hyperresponsiveness after the allergen-induced EAR. L-arginine supplementation reduced the AHR to methacholine and increased iNANC nerve-mediated airway smooth muscle relaxation in guinea pig tracheal preparations, as well as attenuating the AHR to histamine after the EAR in vivo in guinea pigs [18, 47, 49]. Low L-arginine conditions may also lead to increased production of peroxynitrite after the LAR by uncoupling iNOS, allowing it to produce superoxide anions via its reductase domain, which react with NO to form peroxynitrite [84]. Increasing L-arginine availability increases NO production and decreases superoxide and peroxynitrite production in macrophages [85].

## 9. Arginase and Airway Hyperresponsiveness

As arginase plays a role in regulating bioavailability of L-arginine for NOS by competitive consumption of the substrate, increased arginase activity may be responsible for the AHR after the EAR and LAR. In allergen-challenged mice, arginase activity is increased in the airways at the same time as L-arginine and L-citrulline levels are decreased [86]. Arginase's role in allergen-induced AHR is demonstrated by animal studies involving inhibition of both arginase and NOS. Perfused guinea pig tracheal rings treated with specific arginase inhibitor nor-NOHA demonstrated normalization of allergen-induced AHR, and this effect was prevented by coincubation with NOS inhibitor L-NAME, indicating that arginase leads to AHR by decreasing cNOS-derived NO production [42]. iNANC nerve-mediated NO production and smooth muscle relaxation is also restored after the EAR by treatment with nor-NOHA, to a similar level also seen with L-arginine supplementation [47]. Another specific arginase inhibitor (2 (S-) amino-6-boronohexanoic acid or ABH) not only reverses AHR after the EAR and LAR following histamine challenge in a guinea pig model of acute allergic asthma but also prevents AHR when delivered 30 minutes prior to the histamine challenge, most likely related to increased NO production [49]. Similarly, intraperitoneal treatment with nor-NOHA prior to repeated allergen challenge reduced AHR to methacholine in mice [46]. 

As noted above, arginase competition for L-arginine may also contribute to the LAR by increasing peroxynitrite formation through promotion of uncoupling of iNOS under low L-arginine conditions, thereby resulting in the AHR seen after the LAR [57, 87]. Evidence for this relationship comes from the lungs of *D. farinae*-challenged mice which demonstrate increased nitrotyrosine staining and concomitant increased expression of arginase and iNOS [44]. 

Studies in human asthma confirm the importance of arginase in the pathogenesis of experimental asthma. While increased arginase activity in the sputum of asthmatic patients was documented as early as 1980 [88], its role in the pathophysiology of asthma was not further elucidated until decades later. Increased arginase I activity, mRNA, and protein expression have now been demonstrated in inflammatory cells and airway epithelium from bronchial biopsies, as well as bronchoalveolar lavage samples from asthmatic patients [58, 59]. Single nucleotide polymorphisms (SNPs) in both arginase I and arginase II have been associated with atopy, while SNPs in arginase II were associated with increased risk of childhood asthma [89]. Increased arginase activity has also been demonstrated in the serum of asthmatic children experiencing an exacerbation, at the same time as plasma L-arginine levels and the arginine/ornithine ratio (a biomarker that inversely correlates to arginase activity) were reduced. Improvement in asthma symptoms corresponded temporally with reduction of arginase activity and increase in plasma L-arginine levels and the arginine/ornithine ratio [90]. The lung function of severe asthmatics (FEV1 and FEV1/FVC) correlates directly with L-arginine bioavailability, and inversely with serum arginase activity, indicating that serum arginase activity reduces circulating L-arginine levels which contribute to NO deficiency within the airways [91].

## 10. Arginase and Airway Inflammation

Airway inflammation is a key problem in asthma and remains the main therapeutic target for treatment of the disease. Arginase expression has been documented in inflammatory cells of both animal models and humans, but models of arginase inhibition have not yet revealed a consistent effect on inflammatory pathways. Human polymorphonuclear cells and eosinophils constitutively express arginase I, which is located in azurophilic granules and upon release plays a role in regulation of L-arginine concentration, suppression of activation of T-lymphocytes and NK cells, and antimicrobial activity [92]. Arginase is highly expressed in M2 alternatively activated macrophages which are stimulated by IL-4 and IL-13 cytokines produced by CD4+ T_H_2 cells, CD8+ T cells, NK cells, basophils, mast cells, and eosinophils [93, 94]. In contrast, classical activation of macrophages for cytotoxic killing involves a T_H_1 inflammatory pathway in which arginase may play a detrimental role of limiting NO production. Arginase from alternatively activated macrophages, however, may play a role in resolution of inflammation and wound healing via a shift towards synthesis of proline and polyamines instead of NO production [94–96]. 

Contradictory studies of arginase inhibition have reported enhancement, attenuation, and no effect on inflammation in animal models [46, 49, 50, 97] and may reflect issues specific to animal models of asthma in general that often limit our understanding and treatment of asthma [98]. Since chronic asthma is a disease unique to humans, the fact that mice do not have asthma may contribute to the conflicting reports that make the mechanistic translation to human disease more of a challenge. Further studies are needed to clarify these effects and their implications in man. In mice sensitized to ovalbumin, arginase inhibitor S-(2-boronoethyl)-l-cysteine (BEC) increased peribronchiolar and perivascular inflammation associated with increased S-nitrosothiols and 3-nitrotyrosine but did not change allergen-induced increases in differential cell counts or cytokine levels in BAL samples [50]. Unfortunately, the role of low arginine bioavailability and NOS uncoupling as a plausible contributing factor to excess superoxide production in this model is unknown. In another study, arginase inhibitor nor-NOHA administration prior to ovalbumin challenge in mice decreased total cell count in BAL samples by 65% felt to be related to increased NO production, whereas iNOS-knockout mice had an increased inflammatory response to the ovalbumin challenge [46]. ABH administration to guinea pigs similarly inhibited allergen-induced increases in BAL inflammatory cells (eosinophils, macrophages, and total cells) by 50% [49]. Finally, in chimeric mice with arginase I^−/−^ bone marrow, no change was seen in basal or allergen-induced inflammatory cell infiltration or BAL differential cell counts, indicating that at least bone marrow-derived arginase I is not required for development of lung inflammation in the mouse model [97].

## 11. Arginase and Chronic Airway Remodeling

Airway remodeling can be seen in asthma as a result of chronic airway inflammation, and prevention of this complication is a major goal for treatment. Eosinophilia, TGF-*β*, and IL-13 are postulated to play a role in the process of airway remodeling [99]. Arginase may also participate in the development of airway fibrosis via the pathway of L-ornithine production, with subsequent synthesis of polyamines (involved in promotion of cell growth and proliferation) and L-proline (involved in collagen synthesis). Indeed, alternatively activated macrophages expressing arginase I have been implicated in diseases such as idiopathic pulmonary fibrosis [100]. Mouse models of lung fibrosis reveal a dose-dependent correlation between profibrotic factor TGF-*β* and arginase activity in lung tissue and fibroblasts [101]. In mouse models of bleomycin-induced pulmonary fibrosis, increased expression of arginase I and II and decreased L-arginine availability are associated with increased collagen I expression [45]. Inhibition of arginase by nonspecific inhibitor NOHA decreases TGF-*β*1-stimulated collagen deposition [44, 45]. Another mouse model of lung fibrosis investigated the role of TGF-*β* upregulation using transgenic mice expressing human TGF-*β* under the control of the Clara cell promoter. Following treatment with doxycycline, IL-13 was upregulated, macrophages were activated via the alternative pathway, arginase I and II expression and arginase activity were augmented, and pulmonary fibrosis increased [102]. Taken together, arginase seems to play a role in the fibrotic pathway induced by TGF-*β* by metabolism of L-arginine to L-ornithine, leading to downstream production of proline and polyamines, and finally resulting in collagen deposition and fibrosis. 

Arginase may also play a role in airway remodeling and fibrosis by decreasing NO production through competition for L-arginine, thereby reducing antifibrotic and antismooth muscle proliferation effects of NO. Antifibrotic properties of NO are demonstrated in studies of increased allergen-induced collagen deposition in the airways of guinea pigs after treatment with NOS inhibitor L-NAME and increased collagen deposition in allergen-challenged iNOS knockout mice compared to wild type [43, 104]. NO limits proliferation of human and guinea pig cultured airway smooth muscle cells by inhibiting cell cycle progression [105–108]. Finally, arginase may itself contribute to smooth muscle proliferation via synthesis of polyamines and subsequent stimulation of cell proliferation [109–112].

## 12. Arginase and Novel Therapeutic Targets for Asthma

Increased understanding of the role of arginase in the pathogenesis of asthma naturally leads to consideration of novel therapeutic targets for treatment. As noted above, animal models of specific arginase inhibition have demonstrated prevention or reversal of airway hyperresponsiveness associated with allergen challenge, and therefore further development and study of inhaled arginase inhibitors may be a promising area of research. 

Restoration of L-arginine bioavailability to NOS through exogenous supplementation of L-arginine is another potential therapeutic target, although a great deal of orally administered L-arginine is metabolized to urea in the liver. Its utility may be further limited by excess arginase found in seurm during an acute asthma exacerbation in humans [90], skewing metabolism away from NO towards ornithine and its downstream metabolites, proline, and polyamines. Combination therapy utilizing L-arginine with an arginase inhibitor may circumvent this issue. Alternatively, L-citrulline or L-glutamine (converted to citrulline by the enterocytes) could be administered as a prodrug for L-arginine, as citrulline is converted in the kidney to arginine through the “intestinal-renal axis” [113], thus bypassing liver metabolism by arginase. Interestingly, citrulline supplementation in a pilot phase II clinical trial in sickle cell patients resulted in an increase in plasma L-arginine levels [114]. Preliminary results of pharmacokinetic studies using oral L-glutamine have also demonstrated improvement in global arginine bioavailability in patients with SCD and pulmonary hypertension [115].

## 13. Sickle Cell Disease: A Unique Asthma Paradigm

**Figure 1:**
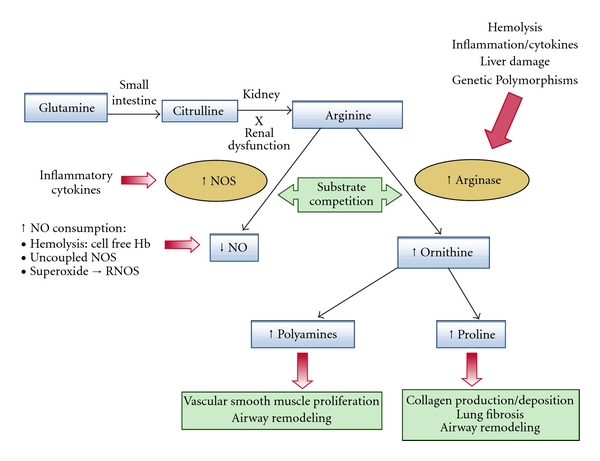
Altered arginine metabolism in hemolysis: a path to pulmonary dysfunction. Dietary glutamine serves as a precursor for the *de novo* production of arginine through the citrulline-arginine pathway. Arginine is synthesized endogenously from citrulline primarily via the intestinal-renal axis. Arginase and nitric oxide synthase (NOS) compete for arginine, their common substrate. In sickle cell disease (SCD) and thalassemia, bioavailability of arginine and NO are decreased by several mechanisms linked to hemolysis. The release of erythrocyte arginase during hemolysis increases plasma arginase levels and shifts arginine metabolism towards ornithine production, limiting the amount of substrate available for NO production. The bioavailability of arginine is further diminished by increased ornithine levels because ornithine and arginine compete for the same transporter system for cellular uptake. Despite an increase in NOS, NO bioavailability is low due to low substrate availability, NO scavenging by cell-free hemoglobin released during hemolysis, and through reactions with free radicals such as superoxide and other reactive NO species. Superoxide is elevated in SCD due to low superoxide dismutase activity, high xanthine oxidase activity, and potentially as a result of uncoupled NOS in an environment of low arginine and/or tetrahydrobiopterin concentration or insufficient NADPH. Endothelial dysfunction resulting from NO depletion and increased levels of the downstream products of ornithine metabolism (polyamines and proline) likely contribute to the pathogenesis of lung injury, pulmonary hypertension, and asthma in SCD. This model has implications for all hemolytic processes as well as pulmonary diseases associated with excess arginase production. This novel disease paradigm is now recognized as an important mechanism in the pathophysiology of SCD and thalassemia. Abnormal arginase activity emerges as a recurrent theme in the pathogenesis of a growing number of diverse pulmonary disorders. Regardless of the initiating trigger, excess arginase activity represents a common pathway in the pathogenesis of asthma and pulmonary hypertension, reproduced with permission from the American Society of Hematology [103].

Sickle cell disease (SCD) is an inherited hemoglobinopathy that causes a chronic hemolytic anemia. Asthma is a common comorbidity in SCD with a reported prevalence of 30–70% [116–124]. The high frequency of asthma in this population cannot be attributed to genetic predisposition alone and likely reflects in part the contribution of overlapping mechanisms shared between these otherwise distinct disorders [90, 125]. There is accumulating evidence that dysregulated arginine metabolism and in particular elevated arginase activity contributes to pulmonary complications in hemolytic disorders that include SCD and the thalassemia syndromes [103, 126–132]. As summarized in this review, derangements of arginine metabolism are also emerging as newly appreciated mechanism in both asthma [57, 90, 91, 133, 134] and pulmonary hypertension independent of SCD [135–139] (Figure 1). Patients with SCD may potentially be at risk for an “asthma-like” condition triggered or worsened by hemolysis-driven release of erythrocyte arginase and low nitric oxide bioavailability [125, 127], in addition to classic familial asthma [140]. An abnormal methacholine challenge and airway hyperreactivity has been documented in up to 78% of children with SCD tested [141–143]. Of interest, a recent study of 99 children with SCD found few typical features of asthma associated with a positive methacholine challenge. Although methacholine responsiveness was correlated to a higher serum IgE, there was no relationship between responsiveness and FEV_1_% predicted, FEV_1_/FVC% predicted, bronchodilatory reactivity, eNO, allergy skin tests, or eosinophil count. However, increased methacholine responsiveness was strongly correlated to higher plasma LDH [143], a biomarker of hemolysis [144] robustly associated with erythrocyte-released plasma arginase concentration [127, 144]. Given the association of asthma with inflammation, oxidative stress and hypoxemia, factors known to contribute to a vasculopathy in SCD, and the consequences of these factors on sickle erythrocytes, comorbid asthma would likely contribute to a vicious cycle of sickling and subsequent complications of SCD [125]. Indeed, a growing body of evidence links asthma to complications in SCD, including acute chest syndrome [145], stroke [120], pulmonary hypertension [124], and early mortality [123]. Whether this is true “asthma” by the conventional definition or an “asthma-like” co-morbidity specific to SCD remains unknown but is a current topic of interest [125, 126, 140, 143]. It is likely that dysregulated arginine metabolism and excess production of proline and polyamines contribute to many forms of abnormal pulmonary function in SCD and other hemoglobinopathies [125]. In fact, we may learn a great deal about the asthma paradigm itself from the “asthma-like” condition that frequently develops in patients with SCD.

## 14. Conclusion

Recent research has revealed mounting evidence for a causal role for arginase in the development of airway hyperresponsiveness, airway inflammation, and chronic airway remodeling which comprise allergic asthma. The effects of arginase on nitric oxide metabolism are related to its competitive consumption of L-arginine, leading to decreased NO production and increased peroxynitrite formation. In addition, the arginase by-product L-ornithine contributes to synthesis of polyamines and L-proline which play a role in pathways of fibrosis by contributing to cellular proliferation and collagen deposition. Further research is warranted to investigate arginase and arginine as potential new therapeutic targets for treatment of asthma.

##  Conflict of Interests

C. R. Morris, MD, is the inventor or coinventor of several Children's Hospital & Research Center Oakland patents/patent applications for biomarkers and novel therapeutics that target global arginine bioavailability; has received research support from Merck; served on scientific advisory committees for Merck and Icagen; received an educational stipend from INO Therapeutics; has been a consultant for Biomarin, Gilead Sciences, Inc, and the Clinical Advisors Independent Consulting Group. K. A. Hardy, MD, is the investigator for several multicenter trials for cystic fibrosis (CF) with funding from the CF Foundation, Inspire pharmaceuticals, and the CDC. She is a member of the speaker bureaus for MedImmune, Glaxo Kline Smith, and Merck. She serves on the asthma advisory boards for Children's First Medical Group and Brown and Toland Medical Group.

## Figures and Tables

**Table 1:** Pharmacologic enzyme inhibitors.

Inhibitor	Specificity	Route	Reference
L-NAME	Nonselective NOS	Oral	[20, 42, 43]
NOHA	Arginase	Inhaled	[39, 44, 45]
nor-NOHA	Arginase	Inhaled, intraperitoneal	[19, 38, 42, 46, 47]
L-NMMA	Arginase	Inhaled	[48]
ABH	Arginase	Inhaled	[49]
BEC	Arginase	Oropharyngeal aspiration	[50]

List of abbreviations: N (G-) nitro-L-arginine methyl ester (L-NAME), nitric oxide synthase (NOS), N-hydroxy-L-arginine (NOHA), N-hydroxy-nor-L-arginine (nor-NOHA), N^G^-monomethyl-L-arginine (L-NMMA), 2(S)-amino-6-boronohexanoic acid (ABH), S-(2-boronoethyl)-l-cysteine (BEC).

**Table 2:** Altered nitric oxide metabolism in allergic asthma.

	Stage of allergic asthma*				
	EAR	LAR	AHR	Inflammation	Remodeling
NO	↓	↑	↑	↑	↓?
cNOS	↓	*↔*	*↔*	*↔*	?
iNOS	*↔*	↑	↑	↑	?
Arginase	↑	↑	↑	?	↑?

*Production of nitric oxide (NO) during different stages of asthma is related to the balance of NOS (nitric oxide synthase) and arginase activity. During the early asthmatic reaction (EAR), increased arginase activity leads to deficiency of L-arginine substrate for cNOS (constitutive NOS), thereby decreasing NO production. iNOS (inducible NOS) is upregulated during the late asthmatic reaction (LAR) leading to increased NO production, at the same time as arginase activity increases, and airway hyperresponsiveness (AHR) increases. The availability of L-arginine substrate for both NOS and arginase may drive reactions and can contribute to proinflammatory peroxynitrite formation under low L-arginine conditions. Elevated NO and iNOS are clearly associated with chronic allergic inflammation, but the role of arginase in this stage is less apparent. At this time, little is known about the roles of NO, NOS, and arginase during airway remodeling in asthma, and further studies are needed to elucidate these pathways.
